# Burden of current and past smoking across 28 European countries in 2017: A cross-sectional analysis

**DOI:** 10.18332/tid/149477

**Published:** 2022-06-14

**Authors:** Ayaka Teshima, Anthony A. Laverty, Filippos T. Filippidis

**Affiliations:** 1 Department of Primary Care and Public Health, School of Public Health, Imperial College London, London, United Kingdom

**Keywords:** tobacco, smoking, tobacco control, epidemiology, socioeconomic status

## Abstract

**INTRODUCTION:**

Most studies use the prevalence of current smoking as an indicator to quantify the burden of smoking. However, length and intensity of smoking, as well as time since cessation for former smokers are also known to impact smoking-related health risks. The aim of this study was to quantify and compare the burden of smoking across the European Union (EU) using a range of smoking burden indicators.

**METHODS:**

We conducted a cross-sectional analysis using data from the March 2017 Eurobarometer 87.1 (n=27901, people aged ≥15 years) in 28 European Union Member States (EU MS) and the Tobacco Control Scale. We defined five indicators of smoking burden including the prevalence of current and ever smoking, length of smoking, pack-years, and discounted pack-years, and ranked EU MS by each indicator. Two-level linear and logistic regressions were performed to assess the association between these indicators and sociodemographic and tobacco policy factors.

**RESULTS:**

Wide variations across the EU countries were observed in all smoking burden indicators. While some MS ranked consistently high (e.g. Greece, France) or consistently low (e.g. Ireland, United Kingdom) in all indicators, we found substantial discrepancies in ranking depending on the indicator used for MS such as Malta, Denmark, Finland and the Netherlands. All indicators of smoking burden were lower among women and respondents without financial difficulties; however, the magnitude of those inequalities varied two-fold among the different indicators.

**CONCLUSIONS:**

Using a range of smoking burden indicators can be more informative than relying on prevalence alone. Our analysis highlights the limitations of relying solely on prevalence of current smoking to estimate the burden of smoking and the potential value of more nuanced indicators. We recommend that multiple and more nuanced indicators that consider former smokers, intensity and duration of smoking should be utilized to monitor tobacco use and evaluate tobacco control policies.

## INTRODUCTION

The tobacco epidemic is a substantial preventable health risk in the EU and it is responsible for about 0.7 million deaths annually^1^. While overall smoking prevalence has fallen in Europe due to tobacco control measures over the past two decades, Europe remains the region with the greatest smoking prevalence globally (29%)^2,3^.

Many of the diseases related to smoking, such as cardiovascular disease, respiratory disease, and malignant neoplasms, develop over long time periods^4-7^. There is a clear dose response relationship, and the mortality risk from cardiovascular events increases with the amount and duration of smoking^8-11^. Several studies show a significant reduction in the incidence of coronary heart disease after cessation, and smoking cessation is beneficial at any stage in reducing excess risk^4,12^. However, there is conflicting evidence on the time needed for the excess risk among former smokers who have quit smoking to revert to the level of health-related risk of never smokers^13-15^. Variability in estimates may be due to differences in methods, the outcomes and the population^13-16^. The majority of studies conclude that the overall mortality risk for former smokers reaches the same level as never smokers 10 to 14 years after quitting^13,15-17^.

Considering the above, estimating the burden of smoking in a population can be challenging as smoking exposure can be quantified by various indicators^7,10,18,19^. Length of smoking and pack-years are well-established measures associated with the incidence and severity of tobacco-related diseases^9,20,21^. Most epidemiological studies use smoking prevalence to quantify the burden of smoking on populations, but smoking intensity/pack-years and former smokers were rarely considered, at least at the population level^22-26^. The most recent tobacco-related estimates of the Global Burden of Disease (GBD) project have incorporated some of these elements. However, GBD focuses on smoking-attributable mortality and disability and does not necessarily provide a direct comparison between these metrics overall or their associations with sociodemographic factors^27^.

Across the EU there are differences in not only smoking prevalence, but also smoking history, proportion of former smokers and smoking intensity. While this is a challenge when trying to make cross-country comparisons, it also provides an opportunity to highlight how various smoking indicators may contribute to our understanding of smoking burden at the country level. To address these evidence gaps, this study aimed to quantify and compare the exposure to smoking across the EU using a wide range of smoking burden indicators and explore how these are associated with sociodemographic and tobacco control factors.

## METHODS

### Data source

We analyzed data from all 27 European countries and the United Kingdom (UK) which was also an EU MS at the time of collection between 18 and 27 March 2017, through the special Eurobarometer 87.1. The Eurobarometer survey used multi-stage sampling: primary sampling units (PSU) proportional to the size of the population were selected from each country region, and standard households were systematically sampled^28^. One person was randomly selected from each household, followed by a face-to-face interview in the participant's home in an appropriate language to collect self-reported data. The Eurobarometer datasets are weighted to guarantee that the samples are nationwide representative with respect to age, sex and area of residence^29^. In total 27901 participants, aged ≥15 years, across the EU MS were surveyed.

### Measures

*Outcome variables*

Five primary outcomes were defined to estimate the burden of smoking at the population level. These are: ‘Prevalence of current smoking’, ‘Prevalence of ever smoking’, ‘Length of smoking’, ‘Pack-years’, and ‘Discounted pack-years’.

*Smoking status*

Participants were asked: ‘Regarding smoking cigarettes, cigars, cigarillos, or a pipe, which of the following applies to you?’. Response options included: ‘You currently smoke’ (current smoking); ‘You used to smoke but you have stopped’ (former smoking); ‘You have never smoked’ (never smoking). We also defined ever smoking as current or former smoking.

*Length of smoking*

All current and former smokers were asked: ‘How old were you when you started smoking regularly, i.e. at least once a week?’. Additionally, former smokers were asked: ‘How old were you when you quit smoking?’. The length of smoking for current smokers was calculated by subtracting the age at which they started smoking regularly from their current age; for former smokers by subtracting the age at which they started from the age they stopped smoking.

*Pack-years*

The number of cigarettes smoked per day for current smokers was assessed using the question: ‘On average, how many cigarettes do you smoke each day?’. The number of cigarettes smoked per day for former smokers was assessed using the question: ‘On average, how many cigarettes did you smoke each day?’. Pack-years of smoking combines smoking duration and smoking intensity. It was calculated by dividing the number of cigarettes per day by 20 (assuming one pack = 20 cigarettes) and multiplying by the number of years of smoking, assuming the average cigarettes per day applies to all previous years.

*Discounted pack-years*

We also developed a novel risk measure of smoking burden based on previous literature which we call ‘discounted pack-years’^4,12,16,30-32^. Discounted pack-years is an attempt to quantify the reduction in smoking risk with time since cessation among former smokers. We assumed that the excess risk for former smokers would be the same as for never smokers after ten years of cessation and the extent of this would decrease linearly over the ten years^16,30^. Therefore, for current smokers, the discounted pack-years are equal to pack-years, and for never smokers, the discounted pack-years are zero. The discounted pack-years for former smokers are calculated by reducing the number of pack-years by 10% for each year of cessation up to ten years, at which point the number of discounted pack-years goes down to zero.

### Covariates

*Sociodemographic data*

The survey collected self-reported demographic data on age (15–24; 25–34; 35–44; 45–54; 55–64; 65–74; ≥75 years), sex (male; female), age at which they stopped full-time education (≤15; 16–19; ≥20 years; still studying), area of residence (rural; small town; large town), occupation (employment; unemployment; not working), marital status (single household without children; single household with children; multiple households without children; multiple households with children), and difficulties to paying bills during the last twelve months (almost never/never; and from time to time/most of the time).

*Tobacco control policies*

Tobacco Control Scale (TCS) scores were used to measure the national-level implementation of tobacco control policies, based on six policies prioritized by the World Bank^33^. The six components of the TCS and their corresponding scores are: tobacco taxes (30 points), smoking bans in public places (22 points), public information campaigns (15 points), advertising ban (13 points), health warning labels (10 points), and cessation support (10 points). We used TCS reports in 2005, 2007, 2010, 2013 and 2016 and calculated the average TCS score (range: 0–100) for each European country as an indicator of tobacco control policies over the 12-year period before the survey. Croatia was only included in 2013 and 2016, hence its score is the average of those two years. Higher scores indicate more comprehensive tobacco control measures were in place. The average TCS scores were divided into three categories: low 1–39.9; moderate 40–49.9; and high ≥50.

### Statistical analysis

We estimated prevalence of current and ever smoking and means of the continuous outcome variables stratified by gender and difficulty paying bills. We present estimates across the entire population, assuming that never smokers had a value of zero in the continuous outcomes: length of smoking, pack-years and discounted pack-years. We also present scatterplots of all possible pairs of country-level estimates for which we also calculated the Pearson r correlation coefficient. Two-level linear regression, accounting for clustering of observations within European countries, was performed for the three continuous outcomes. The models were adjusted for sex, age, age at completion of formal education, financial difficulty, area of residence, occupation, marital status, and country-level TCS scores. Similarly, two-level logistic regression models, adjusted for the same variables were conducted for outcomes prevalence of current and ever smoking. All statistical analyses were performed in Stata Statistical Software: Release 15. Maps were also generated in www.mapchart.net. Observations with missing values and responses of ‘don't know’ were excluded from the analysis. Given the complex survey design, the survey weights included in the original Eurobarometer dataset were used in the descriptive analysis. The study analyzed publicly available, anonymized secondary data and therefore did not require ethical approval from an Institutional Review Board. We present data for prevalence of current smoking, pack-years and discounted pack-years in the main text, whereas results on prevalence of ever smoking and length of smoking are presented in the Supplementary file. We present data for prevalence of current smoking, pack-years and discounted pack-years in the main text, whereas results on prevalence of ever smoking and length of smoking are presented in the Supplementary file.

## RESULTS

The Eurobarometer dataset included a total of 27901 study participants in 28 European countries. The characteristics of the study population and smoking status by sociodemographic characteristics are summarized in Supplementary file Tables 1 and 2. The overall weighted EU prevalence of current smoking was 26.3% (95% CI: 25.4–27.1), with wide variations between countries, ranging from 7.2% in Sweden to 36.6% in Greece. The prevalence of ever smoking across the EU was 46.5% (95% CI: 44.9–48.1), ranging from 37.5% in Ireland to 57.4% in France (Supplementary file Table 3).

**Figure 1:**
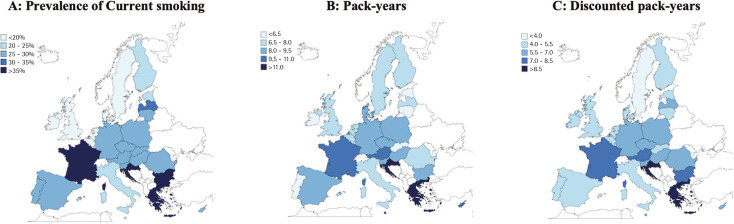
Map of three indicators of smoking burden in the entire population across the EU countries, Eurobarometer 2017

Similarly large differences across the EU were observed in each of the continuous outcomes we assessed (Figure 1 and Supplementary file Figure 1). Greece had the highest population mean of both pack-years (13.9; 95% CI: 12.5–15.2) and discounted pack-years (10.0; 95% CI: 8.9–11.2); Portugal (5.2 pack-years (95% CI: 4.5–6.0) and Sweden (2.2 discounted pack-years (95% CI: 1.6–2.8) had the lowest means (Supplementary file Tables 4–6).

**Figure 2:**
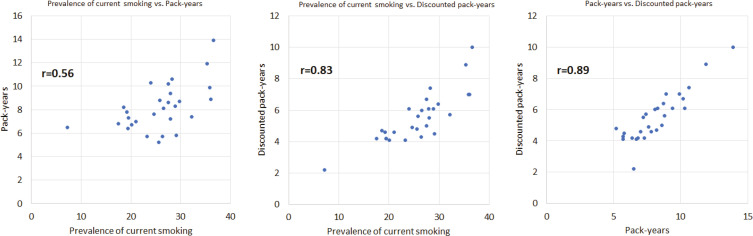
Correlation of prevalence of current smoking, pack-years and discounted pack-years across the EU countries, Eurobarometer 2017

Table 1 shows the prevalence of current smoking, pack-years, and discounted pack-years across the EU. In addition to these three outcomes, Supplementary file Table 7 includes prevalence of ever smoking and length of smoking. Some European countries ranked consistently among the highest (e.g. Greece) or lowest (e.g. Ireland) across the EU in all measures used. However, ranking varied considerably in some of the other European countries. For example, Malta ranked 19th in current and 22nd in ever smokers, but 4th in pack-years and 8th in discounted pack-years. Some major discrepancies in the prevalence of current and ever smoking were also observed. Examples include Denmark (26th in current smokers and 4th in ever smokers), Finland (22nd in current and 8th in ever smokers), and the Netherlands (23rd in current and 5th in ever smokers). Ranking in pack-years and discounted pack-years was somewhat more consistent, but there were exceptions, such as Portugal which ranked 28th in pack-years and 17th in discounted pack-years). This is illustrated in the scatterplots presented in Figure 2 and Supplementary file Figure 2, as well as in the correlation coefficients for different pairs of indicators. For instance, discounted pack-years were strongly correlated to pack years (r=0.89) and prevalence of current smoking (r=0.83), but only weakly correlated to prevalence of ever smoking (r=0.42).

**Table 1:** Three outcomes of smoking burden across the 27 EU countries and the UK, Eurobarometer 2017

*Country*	*Prevalence of current smoking (%)*		*Pack-years*		*Discounted pack-years*	
	*Values and rank*					
Austria	28.3 (25.2–31.6)	**9**	10.6 (9.3–11.8)	**3**	7.4 (6.3–8.4)	**3**
Belgium	19.2 (16.6–22.0)	**25**	7.8 (6.8–8.8)	**15**	4.6 (3.9–5.3)	**19**
Bulgaria	36.1 (33.2–39.2)	**2**	8.9 (8.0–9.8)	**8**	7.0 (6.2–7.8)	**4**
Croatia	35.3 (32.3–38.4)	**4**	11.9 (10.6–13.2)	**2**	8.9 (7.8–10.1)	**2**
Cyprus	27.5 (23.4–32.1)	**12**	10.2 (8.5–11.9)	**5**	6.7 (5.4–7.9)	**6**
Czech Republic	28.9 (26.1–31.9)	**8**	8.3 (7.3–9.2)	**12**	6.1 (5.3–7.0)	**8**
Denmark	18.6 (16.1–21.4)	**26**	8.2 (7.3–9.2)	**13**	4.7 (4.0–5.4)	**18**
Estonia	23.3 (20.4–26.5)	**20**	5.7 (5.0–6.4)	**26**	4.1 (3.5–4.8)	**26**
Finland	20.1 (17.5–23.0)	**22**	6.7 (5.9–7.4)	**22**	4.1 (3.5–4.8)	**26**
France	35.9 (32.6–39.4)	**3**	9.9 (8.8–11.0)	**6**	7.0 (6.1–7.9)	**4**
Germany	25.8 (23.3–28.6)	**16**	8.8 (7.8–9.8)	**9**	5.6 (4.8–6.4)	**13**
Greece	36.6 (33.5–39.8)	**1**	13.9 (12.5–15.2)	**1**	10.0 (8.9–11.2)	**1**
Hungary	26.6 (23.8–29.6)	**14**	8.1 (7.2–9.0)	**14**	6.0 (5.2–6.7)	**11**
Ireland	19.4 (16.9–22.2)	**24**	6.4 (5.6–7.3)	**24**	4.2 (3.5–4.8)	**23**
Italy	24.6 (22.0–27.5)	**18**	7.6 (6.7–8.5)	**16**	4.9 (4.2–5.6)	**16**
Latvia	32.2 (28.4–36.3)	**5**	7.4 (6.2–8.6)	**17**	5.7 (4.6–6.7)	**12**
Lithuania	29.1 (26.0–32.5)	**7**	5.8 (5.1–6.5)	**25**	4.5 (3.9–5.2)	**21**
Luxembourg	21.0 (17.3–25.3)	**21**	7.0 (5.7–8.4)	**20**	4.6 (3.5–5.7)	**19**
Malta	24.0 (19.7–28.9)	**19**	10.3 (8.3–12.3)	**4**	6.1 (4.6–7.5)	**8**
Poland	29.8 (26.9–32.9)	**6**	8.7 (7.7–9.6)	**10**	6.4 (5.5–7.2)	**7**
Portugal	25.6 (23.0–28.4)	**17**	5.2 (4.5–6.0)	**28**	4.8 (4.1–5.5)	**17**
Romania	28.0 (25.2–30.9)	**10**	7.2 (6.2–8.3)	**19**	5.5 (4.6–6.4)	**14**
Slovakia	26.4 (23.5–29.6)	**15**	5.7 (5.0–6.4)	**26**	4.3 (3.7–4.9)	**22**
Slovenia	27.9 (25.0–31.0)	**11**	9.4 (8.3–10.5)	**7**	6.1 (5.2–6.9)	**8**
Spain	27.5 (24.8–30.4)	**12**	8.6 (7.6–9.5)	**11**	5.0 (4.3–5.7)	**15**
Sweden	7.2 (5.3–9.7)	**28**	6.5 (5.4–7.6)	**23**	2.2 (1.6–2.8)	**28**
Netherlands	19.5 (17.0–22.3)	**23**	7.3 (6.5–8.2)	**18**	4.2 (3.5–4.9)	**23**
United Kingdom	17.5 (15.2–20.1)	**27**	6.8 (6.0–7.7)	**21**	4.2 (3.6–4.8)	**23**

**Figure 3:**
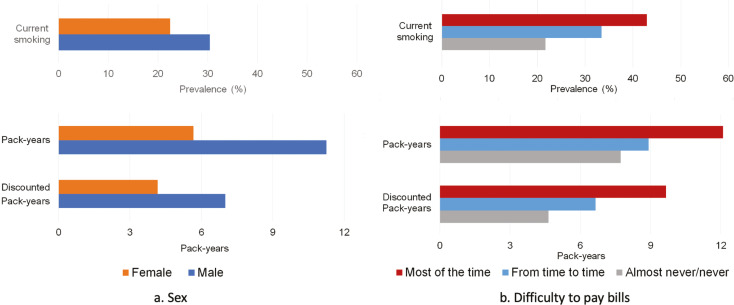
Three indicators of smoking burden by sociodemographic characteristics across the 27 EU countries and the UK

Figure 3 displays the three main outcomes of smoking burden by sex and difficulty paying bills, and the other two outcomes by age and education are shown in Supplementary file Table 8 and Supplementary file Figure 3. Women and respondents without financial difficulties had consistently lower burden of smoking than men and those with financial difficulties. Relative to men, women had lower estimated smoking burden across all indicators; 26% lower in current smoking, 30% lower in ever smoking, 35% lower in length of smoking, 49% lower in pack-years, and 40% lower in discounted pack-years. Furthermore, respondents with no difficulty paying bills had lower smoking burdens than those with financial difficulties: 49% lower in current smoking, 25% lower in ever smoking, 28% lower in length of smoking, 36% lower in pack-years, and 52% lower in discounted pack-years.

In our regression analyses, women were less likely than men to be current smokers (OR=0.57; 95% CI: 0.53–0.60) and had fewer pack-years (β= -7.5; 95% CI: -7.9 – -7.2) and discounted pack-years (β= -4.1; 95% CI: -4.4 – -3.8). Similarly, respondents without financial difficulties had consistently lower burden of smoking compared to those who reported having difficulty paying bills ‘most of the time’. They had lower odds of being current smokers (OR=0.52; 95% CI: 0.47–0.57) and fewer pack years (β= -3.8; 95% CI: -4.5 – -3.4) and discounted pack-years (β= -3.9; 95% CI: -4.5 – -3.4) (Table 2 and Supplementary file Table 9). However, this was not true for TCS scores. Although a higher TCS score was associated with lower prevalence of current smoking (OR=0.69; 95% CI: 0.54–0.87) and lower mean of discounted pack-years (β= -1.2; 95% CI: -2.3–0.0), there was no clear association between TCS score and pack-years (β= -0.5; 95% CI: -2.4–1.5). Findings for other sociodemographic variables, such as occupation, area of residence and education were consistent across all indicators.

**Table 2:** Two-level logistic regression and two-level linear regression estimated associations with three outcomes of smoking burden in the 27 EU countries and the UK, Eurobarometer 2017

*Variables*	*Prevalence of current smoking*	*Pack-years*	*Discounted pack-years*
	*OR (95% CI)^a^*	*β coefficient (95% CI)^b^*	
**Gender**			
Male (Ref.)	1		
Female	0.57 (0.53–0.60)	-7.5 (-7.9 – -7.2)	-4.1 (-4.4 – -3.8)
**Age** (years)			
15–24 (Ref.)	1		
25–34	1.00 (0.86–1.16)	3.1 (2.1–4.2)	3.0 (2.1–3.8)
35–44	0.87 (0.74–1.01)	6.1 (5.0–7.2)	6.3 (5.0–7.6)
45–54	0.86 (0.74–1.01)	9.7 (8.7–10.8)	8.1 (7.2–9.0)
55–64	0.63 (0.54–0.74)	12.4 (11.3–13.5)	8.7 (7.8–9.6)
65–74	0.31 (0.26–0.38)	9.2 (8.0–10.5)	4.7 (3.7–5.7)
≥75	0.13 (0.11–0.17)	6.3 (5.0–7.6)	0.9 (-0.1–2.0)
**Education** (age at completion)			
≤15 years (Ref.)	1		
16–19 years	1.01 (0.92–1.12)	-1.2 (-1.8 – -0.6)	-0.6 (-1.1 – -0.1)
≥20 years	0.61 (0.54–0.67)	-3.7 (-4.4 – -3.1)	-2.8 (-3.4 – -2.3)
Still studying	0.40 (0.32–0.49)	-5.9 (-7.3 – -4.4)	-3.6 (-4.8 – -2.5)
**Difficulty paying bills**			
Most of the time (Ref.)	1		
From time to time	0.76 (0.68–0.84)	-1.9 (-2.7 – -1.2)	-2.1 (-2.7 – -1.5)
Almost never/never	0.52 (0.47–0.57)	-3.8 (-4.5 – -3.0)	-3.9 (-4.5 – -3.4)
**Area of residence**			
Rural (Ref.)	1		
Small town	1.06 (0.99–1.14)	0.0 (-0.4–0.5)	0.2 (-0.2–0.6)
Large town	1.11 (1.03–1.20)	0.5 (0.0–1.0)	0.6 (0.2–1.0)
**Occupation**			
Employed (Ref.)	1		
Unemployed	1.38 (1.24–1.54)	2.3 (1.5–3.1)	2.1 (1.5–2.8)
Not working	1.00 (0.90–1.11)	2.7 (2.0–3.3)	1.2 (0.6–1.7)
**Marital status**			
Single households without children (Ref.)	1		
Single households with children	1.22 (1.08–1.39)	0.4 (-0.5–1.3)	0.0 (-0.7–0.7)
Multiple households without children	0.78 (0.72–0.84)	-0.9 (-1.4 – -0.4)	-1.6 (-2.0 – -1.2)
Multiple households with children	0.67 (0.62–0.73)	-1.3 (-1.9 – -0.8)	-2.0 (-2.5 – -1.6)
**TCS score**			
Low (Ref.)	1		
Moderate	0.94 (0.76–1.17)	-0.5 (-2.2–1.2)	-0.5 (-1.5–0.6)
High	0.69 (0.54–0.87)	-0.5 (-2.4–1.5)	-1.2 (-2.3–0.0)

TCS: tobacco control scale.

a Two-level logistic regression adjusted for all variables included in the table.

b Two-level linear regression adjusted for all variables included in the table.

## DISCUSSION

This analysis found wide variation across the EU in the burden of smoking using a range of measures, including prevalence of current and ever smoking but also length of smoking, pack-years, and discounted pack-years. Differences between EU countries, but also by sex and socioeconomic status varied depending on the measure used. Country-level higher TCS scores were only associated with lower smoking prevalence and lower discounted pack-years.

Smoking prevalence is often used as the main measure to describe smoking and the associated potential health burden in a country^1,22,34,35^, however, the results of this study suggest that this may not always be the most appropriate approach to estimate smoking burden. Although prevalence of current smoking is a good measure of the proportion of the population that is exposed to the harmful effects of smoking, it ignores former smokers who may still be at higher risk of smoking-related diseases^4,5,21^. The number of former smokers varies widely between countries that are at different stages of the tobacco epidemic, hence international comparisons based solely on current prevalence may lead to false conclusions^3^. For instance, our analysis identified several countries, such as Denmark, Sweden, the Netherlands and Finland where current smokers are only a fraction of ever smokers, whereas in others, such as Romania and Hungary, current smokers easily outnumber former smokers.

Considering that the prevalence of former or/and ever smoking can address some of these concerns, this may not be sufficient for two reasons. The first is the dose response association between smoking and its health effects and the second is the gradual reduction of risk –relative to never smokers – following smoking cessation^7,9,11,12^. Measuring the mean length of smoking in the population can provide a rough estimate of the average exposure to smoking-related harms in the population, but more detailed measures, such as pack-years and discounted pack-years are more informative as they also consider smoking intensity. Although there are some EU MS which ranked consistently high (e.g. Greece, France) or low (e.g. Ireland, the UK) in all indicators, our analysis clearly illustrates the value of the more detailed indicators, as smoking intensity and duration differ considerably between EU countries. For instance, the prevalence of current and ever smoking is relatively low in Malta, but due to the high consumption of cigarettes among smokers, the actual burden of smoking ranked among the highest in the EU when considering pack-years and discounted pack-years. Lithuania was on the other end of the spectrum with high prevalence of current smoking, but among the less affected EU countries in terms of mean pack-years.

These differences between the indicators used in this study can be seen in the comparisons by sex and financial difficulties. Sex^36-38^ and socioeconomic^39-41^ inequalities in smoking are well established in Europe and can be attributed to differences in patterns of smoking initiation, intensity, and cessation^19,42^. All our indicators were successful in identifying that the burden of smoking was substantially lower in women compared to men, but the magnitude of these differences depended largely on the indicator. Men across the EU smoke more, start younger and quit at an older age than women; there are also more male former smokers, especially among older age groups^35^. Therefore, prevalence of current smoking may overestimate the total smoking-related burden among women compared to men. In our analysis, women had 26% lower prevalence of current smoking than men, but 49% and 40% lower mean pack-years and discounted pack-years, respectively. Similarly, the disparity between those with and without financial difficulties was almost twice as large when considering current smoking or discounted pack-years compared to ever smoking, which reflects the higher quitting rates observed among those in higher socioeconomic groups^19,42,43^.

Among the five indicators, prevalence of current smoking is the most straightforward and easy to measure, which likely explains why it is universally used. In contrast, discounted pack-years, the most nuanced of the indicators, takes into account the length and intensity of smoking, as well as the time since cessation, which are important factors when considering the potential health impact of smoking on individuals and at the population level^7,9^. However, it has to be acknowledged that it requires much more information which may not always be available, including details about the individual’s smoking history. In our analysis, we made some key assumptions that may not always be true, such as that excess risk declines linearly and current average cigarette consumption accurately reflects historical consumption. Obtaining detailed smoking history may not be feasible in large population studies. Both prevalence of current smoking and discounted pack-years led to similar conclusions in several of our analyses, including the association between tobacco control policies and burden of smoking. Nevertheless, it is clear that a more nuanced indicator may be more appropriate in some cases, as illustrated in comparing men and women.

In our analysis, we refer to burden of smoking not only in terms of mortality and morbidity already caused by smoking, but also with regard to the future risk of current and former smokers. Estimates of deaths and Disability Adjusted Life Years (DALYs), such as those produced by the GBD project are valuable, and this study adds information on the overall exposure of the population to smoking, which may impact their health in the near future.

### Strengths and limitations

Although Eurobarometer does not release the survey’s response rate, it provides weights to ensure that the sample is representative of the EU population aged ≥15 years and sampling methods and questionnaires are standardized across the European countries. Thus, it enables valid cross-country comparisons and generalization of results across the EU population. This consistency allowed us to explore a range of indicators to measure the burden of smoking in the EU and utilize inherent differences between EU countries to identify advantages and disadvantages of each method. All data were self-reported, which may have resulted in inaccuracies, especially with regard to information about the distant past, such as past cigarette consumption and age of smoking initiation. Eurobarometer questions do not define a timeframe for smoking abstinence, therefore the definition of former smokers may be ambiguous. However, we have no reason to believe that these inaccuracies have had a differential impact across countries.

This analysis was only able to assess people who use cigarettes. Alternative products such as e-cigarettes and heated tobacco products have gained increasing market share in recent years^44,45^ complicating any estimates of smoking-related burden, as their long-term health risks for exclusive and dual users are still unclear. Finally, our measure of discounted pack-years is mostly based on risk assumptions from studies in the US, and the exact time required for mortality risks of former smokers to reach those of never smokers remains debatable and may vary by type of smokingrelated disease^13-15^.

## CONCLUSIONS

Substantial differences between EU countries exist not only in smoking prevalence, but also in length of smoking, pack-years, and discounted pack-years. Our results suggest that measuring smoking burden by current smoking prevalence alone may be inadequate and lead to under- or over-estimation of the true health risks associated with smoking. Using multiple indicators which take into account past length and intensity of smoking, as well as time since cessation could provide a more accurate picture to forecast future health burden for researchers and policy makers, although collection of the appropriate data may be challenging.

## Data Availability

The data supporting this research are available from the following sources: ZA6861 doi:10.4232/1.1292, available from https://dbk.gesis.org/dbksearch/sdesc2.asp?no=6861&db=e&doi=10.4232/1.12915.
