# Supplementary material for: Burden of current and past smoking across 28 European countries in 2017: A cross-sectional analysis
Source: Tob Induc Dis. 2022 Jun 14;20:56. doi: 10.18332/tid/149477 (PMC9194927; doi:10.18332/tid/149477)
Supplement: Supplementary file 1 [file TID-20-56-s1.pdf]

Supplementary material

Supplementary Table 1. Baseline Participant Characteristics in the 27 EU countries and the UK, Eurobarometer 2017

Characteristics [a]	Entire Sample (n=27,901)
	N, (weighted %)
**Smoking status**	
Current smokers	6904 (26.3 %)
Former smokers	6153 (20.3 %)
Never smokers	14783 (53.5 %)
**Gender**	
Female	15338 (51.8 %)
Male	12563 (48.2 %)
**Age**	
15-24 years	2463 (13.0 %)
25-34 years	3619 (14.8 %)
35-44 years	4216 (16.3 %)
45-54 years	4533 (17.1 %)
55-64 years	5177 (14.7 %)
65-74 years	4998 (15.2 %)
≥75 years	2895 (8.9 %)
**Education (age at completion)**	
≤15 years	4260 (17.2 %)
16-19 years	11898 (42.0 %)
≥20 years	9567 (31.5 %)
Still studying	1748 (9.4 %)
**Difficulty to pay bills**	
Most of the time	2642 (8.4 %)
From time to time	6967 (25.2 %)
Almost never/never	17827 (66.4 %)
**Area of residence**	
Rural	9089 (30.3 %)
Small town	11087 (44.8 %)
Large town	7709 (24.9 %)
**Occupation**	
Employment	14895 (55.7 %)
Unemployment	1867 (6.9 %)
Not working	11139 (37.4 %)
**Marital Status**	
Single households without children	8282 (30.6 %)
Single households with children	1559 (5.7 %)
Multiple households without children	9421 (31.6 %)
Multiple households with children	8387 (32.1 %)

Supplementary Table 2: Smoking status by socio-demographic characteristics in the 27 EU countries and the UK, Eurobarometer 2017. (N=27,901)

Characteristics [a]	Current smokers, weighted (95%CI) [b]	Former smokers, weighted (95%CI)	Never smokers, weighted (95%CI)
**Gender (%)**			
Male	30.4 (29.0 - 31.7)	24.8 (23.7 - 26.0)	44.8 (43.4 - 46.3)
Female	22.4 (21.3 - 23.6)	16.0 (15.1 - 17.0)	61.5 (60.2 - 62.8)
**Age (%)**			
15 - 24 years	29.6 (26.8 - 32.5)	6.4 (5.0 - 8.1)	64.0 (61.0 - 67.0)
25 - 34 years	32.9 (30.5 - 35.4)	13.8 (12.1 - 15.6)	53.3 (50.8 - 55.9)
35 - 44 years	30.8 (28.5 - 33.2)	18.5 (16.7 - 20.5)	50.7 (48.2 - 53.2)
45 - 54 years	31.5 (29.3 - 33.9)	20.4 (18.4 - 22.5)	48.1 (45.6 - 50.6)
55 - 64 years	27.1 (25.1 - 29.1)	27.4 (25.4 - 29.5)	45.5 (43.3 - 47.8)
65 - 74 years	16.2 (14.5 - 18.0)	29.7 (27.7 - 31.9)	54.1 (51.7 - 56.4)
≥75 years	7.9 (6.5 - 9.5)	26.4 (23.9 - 29.0)	65.7 (62.9 - 68.4)
**Education (Age at completion) (%)**			
≤15 years	22.9 (21.0 - 24.9)	22.6 (20.8 - 24.6)	54.5 (52.2 - 56.8)
16–19 years	32.2 (30.8 - 33.7)	20.6 (19.4 - 21.9)	47.1 (45.6 - 48.7)
≥20 years	21.8 (20.4 - 23.3)	23.2 (21.8 - 24.6)	55.1 (53.4 - 56.8)
Still studying	22.3 (19.3 - 25.5)	5.8 (4.4 - 7.8)	71.9 (68.4 - 75.1)
**Difficulty to pay bills (%)**			
Most of the time	42.9 (39.7 - 46.2)	15.9 (13.7 - 18.5)	41.2 (38.1 - 44.3)
From time to time	33.4 (31.6 - 35.3)	17.2 (15.8 - 18.7)	49.4 (47.5 - 51.3)
Almost never/never	21.7 (20.7 - 22.7)	22.1 (21.1 - 23.1)	56.2 (55.0 - 57.4)
**Area of residence (%)**			
Rural	24.8 (23.3 - 26.3)	20.1 (18.8 - 21.5)	55.1 (53.3 - 56.8)
Small town	26.9 (25.5 - 28.3)	21.2 (20.1 - 22.5)	51.9 (50.3 - 53.4)
Large town	26.8 (25.2 - 28.4)	18.8 (17.4 - 20.2)	54.4 (52.6 - 56.3)
**Occupation (%)**			
Employment	28.9 (27.7 - 30.1)	18.2 (17.2 - 19.3)	52.9 (51.6 - 54.2)
Unemployment	46.5 (42.7 - 50.4)	16.6 (14.1 - 19.5)	36.8 (33.2 - 40.6)
Not working	18.6 (17.4 - 19.9)	24.0 (22.7 - 25.3)	57.4 (55.8 - 58.9)
**Marital Status (%)**			
Single households without children	28.4 (26.8 - 30.1)	16.1 (15 .0- 17.4)	55.5 (53.7 - 57.2)
Single households with children	37.1 (33.1 - 41.3)	18.6 (15.6 - 22.0)	44.3 (40.2 - 48.5)
Multiple households without children	22.5 (21.1 - 23.9)	24.9 (23.5 - 26.3)	52.6 (50.9 - 54.2)
Multiple households with children	26.3 (24.7 - 27.9)	20.1 (18.7 - 21.6)	53.6 (51.8 - 55.4)

Footnote:
a. Summary statistics are displayed as N (%) for categorical variables.
b. CI= Confidence interval.
All the results are weighted

Supplementary Table 3. Smoking prevalence by each smoking status in the 27 EU countries and the UK, Eurobarometer 2017. (N=27,901)

Country	Current smokers (%), weighted (95% CI)	Former smokers (%), weighted (95%CI)	Never smokers (%), weighted (95%CI)
Austria	28.3 (25.2 - 31.6)	18.7 (16.2 - 21.4)	53.0 (49.6 - 56.4)
Belgium	19.2 (16.6 - 22.0)	24.0 (21.3 - 27.1)	56.8 (53.3 - 60.2)
Bulgaria	36.1 (33.2 - 39.2)	12.4 (10.5 - 14.5)	51.5 (48.3 - 54.6)
Croatia	35.3 (32.3 - 38.4)	15.5 (13.2 - 18.0)	49.3 (46.0 - 52.5)
Cyprus	27.5 (23.4 - 32.1)	17.4 (14.3 - 21.0)	55.1 (50.3 - 59.8)
Czech Republic	28.9 (26.1 - 31.9)	18.6 (16.3 - 21.2)	52.4 (49.3 - 55.6)
Denmark	18.6 (16.1 - 21.4)	32.9 (29.9 - 36.1)	48.5 (45.0 - 52.0)
Estonia	23.3 (20.4 - 26.5)	23.9 (21.1 - 27.0)	52.8 (49.3 - 56.2)
Finland	20.1 (17.5 - 23.0)	29.1 (26.2 - 32.2)	50.8 (47.5 - 54.1)
France	35.9 (32.6 - 39.4)	21.5 (18.9 - 24.4)	42.5 (39.1 - 46.0)
Germany	25.8 (23.3 - 28.6)	21.0 (18.7 - 23.5)	53.2 (50.2 - 56.2)
Greece	36.6 (33.5 - 39.8)	19.1 (16.7 - 21.8)	44.3 (41.1 - 47.6)
Hungary	26.6 (23.8 - 29.6)	14.5 (12.5 - 16.8)	58.9 (55.6 - 62.0)
Ireland	19.4 (16.9 - 22.2)	18.1 (15.8 - 20.6)	62.5 (59.3 - 65.6)
Italy	24.6 (22.0 - 27.5)	13.7 (11.6 - 16.0)	61.7 (58.5 - 64.8)
Latvia	32.2 (28.4 - 36.3)	22.7 (19.4 - 26.5)	45.1 (41.0 - 49.2)
Lithuania	29.1 (26.0 - 32.5)	18.0 (15.6 - 20.8)	52.9 (49.4 - 56.3)
Luxembourg	21.0 (17.3 - 25.3)	22.2 (18.6 - 26.2)	56.8 (52.1 - 61.4)
Malta	24.0 (19.7 - 28.9)	19.0 (15.6 - 23.0)	57.0 (51.9 - 61.9)
Poland	29.8 (26.9 - 32.9)	17.7 (15.3 - 20.2)	52.5 (49.2 - 55.8)
Portugal	25.6 (23.0 - 28.4)	14.1 (12.1 - 16.3)	60.3 (57.3 - 63.3)
Romania	28.0 (25.2 - 30.9)	14.0 (11.8 - 16.5)	58.1 (54.8 - 61.2)
Slovakia	26.4 (23.5 - 29.6)	16.7 (14.4 - 19.2)	56.9 (53.5 - 60.1)
Slovenia	27.9 (25.0 - 31.0)	19.4 (17.0 - 22.0)	52.7 (49.5 - 55.9)
Spain	27.5 (24.8 - 30.4)	22.3 (19.8 - 25.0)	50.2 (47.1 - 53.4)
Sweden	7.2 (5.3 - 9.7)	40.6 (36.5 - 45.0)	52.2 (47.8 - 56.5)
Netherlands	19.5 (17.0 - 22.3)	31.7 (28.8 - 34.8)	48.8 (45.4 - 52.1)
United Kingdom	17.5 (15.2 - 20.1)	22.5 (20.0 - 25.2)	60.0 (56.8 - 63.1)

Supplementary Table 4: Mean length of smoking by each smoking status across the 27 EU countries and the UK, Eurobarometer 2017. (N=27,901)

	Length of smoking (Years), weighted (95%CI)			
**Country**	**Ever smokers**	**Current smokers**	**Former smokers**	**Entire sample**
Austria	23.4 (22.0 - 24.9)	25.1 (23.1 - 27.2)	20.8 (18.7 - 22.9)	10.6 (9.6 - 11.6)
Belgium	23.1 (21.7 - 24.6)	25.7 (23.5 - 27.8)	21.1 (19.2 - 22.9)	9.9 (9.0 - 10.8)
Bulgaria	23.3 (22.1 - 24.5)	23.2 (21.8 - 24.7)	23.6 (21.3 - 25.8)	10.9 (10.0 - 11.8)
Croatia	24.9 (23.4 - 26.3)	25.7 (23.9 - 27.5)	22.8 (20.4 - 25.2)	12.0 (10.9 - 13.1)
Cyprus	20.6 (18.9 - 22.3)	21.6 (19.2 - 24.1)	19.0 (16.6 - 21.4)	9.2 (8.1 - 10.4)
Czech Republic	23.2 (21.8 - 24.7)	24.5 (22.5 - 26.6)	21.1 (19.1 - 23.0)	10.7 (9.7 - 11.7)
Denmark	24.6 (23.1 - 26.1)	31.1 (28.2 - 34.0)	21.0 (19.4 - 22.6)	12.6 (11.6 - 13.6)
Estonia	20.4 (18.9 - 21.8)	25.6 (23.4 - 27.7)	15.3 (13.6 - 16.9)	9.5 (8.7 - 10.4)
Finland	20.8 (19.5 - 22.2)	26.9 (24.6 - 29.2)	16.6 (15.2 - 18.1)	10.1 (9.2 - 11.0)
France	23.9 (22.6 - 25.2)	25.3 (23.5 - 27.1)	21.6 (19.8 - 23.4)	13.7 (12.6 - 14.8)
Germany	23.1 (21.8 - 24.3)	25.4 (23.7 - 27.2)	20.0 (18.4 - 21.7)	10.6 (9.8 - 11.5)
Greece	25.0 (23.7 - 26.3)	26.0 (24.3 - 27.7)	23.1 (21.0 - 25.2)	13.9 (12.8 - 15.0)
Hungary	23.4 (21.9 - 24.9)	24.6 (22.5 - 26.6)	21.2 (19.0 - 23.3)	9.5 (8.7 - 10.4)
Ireland	22.3 (20.8 - 23.7)	23.9 (21.5 - 26.2)	20.5 (18.7 - 22.3)	8.3 (7.5 - 9.1)
Italy	27.0 (25.5 - 28.6)	27.8 (25.8 - 29.8)	25.6 (23.3 - 28.0)	10.1 (9.0 - 11.1)
Latvia	21.2 (19.6 - 22.9)	23.6 (21.5 - 25.8)	17.7 (15.2 - 20.2)	11.6 (10.3 - 12.8)
Lithuania	20.7 (19.2 - 22.2)	23.0 (21.1 - 25.0)	16.9 (14.8 - 19.0)	9.6 (8.7 - 10.5)
Luxembourg	20.3 (18.4 - 22.3)	23.3 (20.3 - 26.2)	17.5 (15.0 - 20.0)	8.6 (7.4 - 9.8)
Malta	23.0 (20.9 - 25.1)	23.9 (20.9 - 26.9)	21.7 (18.8 - 24.7)	9.8 (8.4 - 11.1)
Poland	23.3 (21.8 - 24.8)	25.7 (23.7 - 27.6)	19.1 (16.9 - 21.2)	10.7 (9.7 - 11.7)
Portugal	22.5 (21.1 - 23.9)	22.9 (21.1 - 24.7)	21.8 (19.7 - 23.9)	8.8 (7.9 - 9.6)
Romania	22.4 (20.8 - 23.9)	22.8 (20.9 - 24.8)	21.4 (19.0 - 23.9)	9.1 (8.1 - 10.1)
Slovakia	20.3 (18.8 - 21.7)	22.2 (20.2 - 24.3)	17.2 (15.3 - 19.0)	8.5 (7.6 - 9.3)
Slovenia	22.5 (21.3 - 23.8)	24.9 (23.1 - 26.7)	19.2 (17.5 - 20.9)	10.5 (9.6 - 11.4)
Spain	23.4 (22.2 - 24.7)	24.6 (22.8 - 26.3)	22.1 (20.3 - 23.9)	11.6 (10.7 - 12.5)
Sweden	21.0 (19.2 - 22.8)	35.2 (29.8 - 40.6)	18.6 (16.8 - 20.3)	9.9 (8.7 - 11.2)
Netherlands	22.9 (21.5 - 24.3)	29.0 (26.3 - 31.7)	19.2 (17.8 - 20.6)	11.7 (10.7 - 12.7)
United Kingdom	23.4 (21.8 - 25.0)	27.0 (24.2 - 29.7)	20.6 (18.8 - 22.4)	9.3 (8.4 - 10.2)
**EU 28**	**23.6 (23.2 – 24.0)**	**25.6 (25.0 - 26.3)**	**21.0 (20.4 - 21.5)**	**10.8 (10.5 - 11.1)**

Footnote:

CI= Confidence interval.
All the results are weighted.

Supplementary Table 5: Mean pack-years by each smoking status across the 27 EU countries and the UK, Eurobarometer 2017. (N=27,901)

	**Pack-years, weighted (95%CI)**			
**Country**	**Ever smokers**	**Current smokers**	**Former smokers**	**Entire population**
Austria	23.7 (21.6 - 25.8)	24.0 (21.4 - 26.7)	23.2 (19.6 - 26.7)	10.6 (9.3 - 11.8)
Belgium	19.1 (17.0 - 21.2)	17.8 (15.5 - 20.2)	20.2 (17.0 - 23.5)	7.8 (6.8 - 8.8)
Bulgaria	19.3 (17.8 - 20.7)	18.9 (17.2 - 20.5)	20.5 (17.8 - 23.2)	8.9 (8.0 - 9.8)
Croatia	24.8 (22.8 - 26.9)	23.9 (21.7 - 26.1)	27.1 (22.5 - 31.7)	11.9 (10.6 - 13.2)
Cyprus	23.0 (19.9 - 26.2)	20.7 (17.3 - 24.2)	26.7 (20.8 - 32.6)	10.2 (8.5 - 11.9)
Czech Republic	18.2 (16.5 - 19.8)	19.2 (17.0 - 21.3)	16.6 (14.0 - 19.2)	8.3 (7.3 - 9.2)
Denmark	16.7 (15.1 - 18.3)	20.1 (17.1 - 23.1)	14.8 (13.0 - 16.6)	8.2 (7.3 - 9.2)
Estonia	12.4 (11.2 - 13.7)	16.2 (14.2 - 18.3)	8.7 (7.3 - 10.1)	5.7 (5.0 - 6.4)
Finland	14.0 (12.6 - 15.3)	17.3 (15.0- 19.6)	11.6 (10.1 - 13.2)	6.7 (5.9 - 7.4)
France	17.9 (16.2 - 19.5)	16.1 (14.2 - 17.9)	21.2 (18.1 - 24.3)	9.9 (8.8 - 11.0)
Germany	19.5 (17.7 - 21.3)	19.5 (17.2 - 21.8)	19.5 (16.6 - 22.4)	8.8 (7.8 - 9.8)
Greece	25.3 (23.4 - 27.3)	23.9 (21.7 - 26.2)	28.2 (24.4 - 31.9)	13.9 (12.5 - 15.2)
Hungary	20.1 (18.3 - 22.0)	20.2 (18.0 - 22.3)	20.1 (16.6 - 23.6)	8.1 (7.2 - 9.0)
Ireland	17.7 (15.9 - 19.6)	16.6 (14.4 - 18.8)	19.1 (16.0 - 22.3)	6.4 (5.6 - 7.3)
Italy	20.7 (19.0 - 22.4)	19.0 (17.1 - 20.9)	23.9 (20.9 - 27.0)	7.6 (6.7 - 8.5)
Latvia	13.9 (12.0 - 15.9)	13.6 (11.9 - 15.3)	14.4 (10.1 - 18.6)	7.4 (6.2 - 8.6)
Lithuania	12.8 (11.5 - 14.1)	14.3 (12.7 - 15.9)	10.0 (7.8 - 12.2)	5.8 (5.1 - 6.5)
Luxembourg	17.2 (14.4 - 20.1)	18.1 (14.4 - 21.7)	16.4 (12.0 - 20.8)	7.0 (5.7 - 8.4)
Malta	24.4 (20.4 - 28.5)	20.5 (16.3 - 24.7)	29.4 (22.1 - 36.8)	10.3 (8.3 - 12.3)
Poland	19.2 (17.5 - 20.9)	20.3 (18.2 - 22.5)	17.1 (14.3 - 19.8)	8.7 (7.7 - 9.6)
Portugal	17.4 (15.4 - 19.3)	16.5 (14.7 - 18.2)	29.9 (16.7 - 43.1)	5.2 (4.5 - 6.0)
Romania	18.3 (16.2 - 20.4)	18.4 (16.0 - 20.8)	18.1 (13.8 - 22.3)	7.2 (6.2 - 8.3)
Slovakia	13.8 (12.5 - 15.2)	14.9 (13.1 - 16.6)	12.1 (10.0 - 14.2)	5.7 (5.0 - 6.4)
Slovenia	20.4 (18.4 - 22.4)	20.2 (17.8 - 22.6)	20.7 (17.4 - 24.1)	9.4 (8.3 - 10.5)
Spain	17.6 (16.0 - 19.2)	15.1 (13.3 - 16.9)	20.6 (17.8 - 23.3)	8.6 (7.6 - 9.5)
Sweden	14.2 (12.2 - 16.3)	15.7 (11.5 - 19.8)	14.0 (11.7 - 16.3)	6.5 (5.4 - 7.6)
Netherlands	15.1 (13.6 - 16.6)	16.9 (14.2 - 19.7)	13.9 (12.2 - 15.7)	7.3 (6.5 - 8.2)
United Kingdom	17.6 (15.7 - 19.5)	17.4 (14.9 - 19.9)	17.8 (15.1 - 20.5)	6.8 (6.0 - 7.7)
**EU 28**	**18.6 (18.1 - 19.2)**	**18.2 (17.6 - 18.9)**	**19.2 (18.3 - 20.1)**	**8.3 (8.0 - 8.6)**

Supplementary Table 6: Mean discounted pack-years by each smoking status across the 27 EU countries and the UK, Eurobarometer 2017. (N=27,901)

	**Discounted pack-years, weighted (95%CI)**			
**Country**	**Ever smokers**	**Current smokers**	**Former smokers**	**Entire population**
Austria	16.5 (14.5 - 18.5)	24.0 (21.4 - 26.7)	4.6 (2.7 - 6.5)	7.4 (6.3 - 8.4)
Belgium	11.3 (9.7 - 12.8)	17.8 (15.5 - 20.2)	5.4 (3.7 - 7.1)	4.6 (3.9 - 5.3)
Bulgaria	15.1 (13.7 - 16.5)	18.9 (17.2 - 20.5)	3.9 (2.6 - 5.3)	7.0 (6.2 - 7.8)
Croatia	18.7 (16.8 - 20.6)	23.9 (21.7 - 26.1)	5.9 (3.5 - 8.3)	8.9 (7.8 - 10.1)
Cyprus	15.0 (12.6 - 17.5)	20.7 (17.3 - 24.2)	5.9 (3.4 - 8.5)	6.7 (5.4 - 7.9)
Czech Republic	13.4 (11.9 - 15.0)	19.2 (17.0 - 21.3)	4.5 (3.0 - 5.9)	6.1 (5.3 - 7.0)
Denmark	9.5 (8.2 - 10.8)	20.1 (17.1 - 23.1)	3.5 (2.7 - 4.3)	4.7 (4.0 - 5.4)
Estonia	9.0 (7.8 - 10.2)	16.2 (14.2 - 18.3)	1.9 (1.2 - 2.6)	4.1 (3.5 - 4.8)
Finland	8.7 (7.4 - 9.9)	17.3 (15.0 - 19.6)	2.6 (1.8 - 3.4)	4.1 (3.5 - 4.8)
France	12.6 (11.2 - 14.0)	16.1 (14.2 - 17.9)	6.2 (4.2 - 8.1)	7.0 (6.1 - 7.9)
Germany	12.5 (10.9 - 14.0)	19.5 (17.2 - 21.8)	3.9 (2.7 - 5.0)	5.6 (4.8 - 6.4)
Greece	18.3 (16.5 - 20.1)	23.9 (21.7 - 26.2)	6.5 (4.7 - 8.3)	10.0 (8.9 - 11.2)
Hungary	14.9 (13.4 - 16.5)	20.2 (18.0 - 22.3)	4.8 (3.1 - 6.4)	6.0 (5.2 - 6.7)
Ireland	11.5 (10.0 - 13.1)	16.6 (14.4 - 18.8)	5.5 (3.5 - 7.5)	4.2 (3.5 - 4.8)
Italy	13.4 (11.8 - 14.9)	19.0 (17.1 - 20.9)	3.0 (1.9 - 4.1)	4.9 (4.2 - 5.6)
Latvia	10.7 (8.9 - 12.4)	13.6 (11.9 - 15.3)	6.0 (2.6 - 9.5)	5.7 (4.6 - 6.7)
Lithuania	10.1 (8.8 - 11.3)	14.3 (12.7 - 15.9)	2.2 (1.3 - 3.0)	4.5 (3.9 - 5.2)
Luxembourg	11.2 (8.9 - 13.5)	18.1 (14.4 - 21.7)	4.3 (2.2 - 6.3)	4.6 (3.5 - 5.7)
Malta	14.4 (11.4 - 17.3)	20.5 (16.3 - 24.7)	6.6 (2.8 - 10.3)	6.1 (4.6 - 7.5)
Poland	14.1 (12.5 - 15.7)	20.3 (18.2 - 22.5)	2.9 (1.8 - 3.9)	6.4 (5.5 - 7.2)
Portugal	15.8 (14.1 - 17.6)	16.5 (14.7 - 18.2)	7.3 (0.1 - 14.4)	4.8 (4.1 - 5.5)
Romania	13.9 (11.9 - 15.8)	18.4 (16.0 - 20.8)	3.8 (2.2 - 5.3)	5.5 (4.6 - 6.4)
Slovakia	10.4 (9.1 - 11.7)	14.9 (13.1 - 16.6)	3.2 (1.9 - 4.4)	4.3 (3.7 - 4.9)
Slovenia	13.2 (11.6 - 14.9)	20.2 (17.8 - 22.6)	3.3 (2.0 - 4.6)	6.1 (5.2 - 6.9)
Spain	10.2 (9.0 - 11.4)	15.1 (13.3 - 16.9)	4.1 (3.0 - 5.3)	5.0 (4.3 - 5.7)
Sweden	4.7 (3.5 - 6.0)	15.7 (11.5 - 19.8)	2.8 (1.6 - 4.0)	2.2 (1.6 - 2.8)
Netherlands	8.6 (7.3 - 9.9)	16.9 (14.2 - 19.7)	3.6 (2.5 - 4.7)	4.2 (3.5 - 4.9)
United Kingdom	10.8 (9.4 - 12.3)	17.4 (14.9 - 19.9)	5.4 (3.9 - 6.9)	4.2 (3.6 - 4.8)
**EU 28**	**12.4 (11.9 – 12.8)**	**18.2 (17.6 - 18.9)**	**4.3 (3.9 - 4.8)**	**5.5 (5.3 - 5.8)**

Supplementary Table 7. Five outcomes of smoking burden across the 27 EU countries and the UK, Eurobarometer 2017

Country	Prevalence of current smoking (%)		Prevalence of ever smoking (%)		Length of smoking (years)		Pack-years (pack-years)		Discounted Pack-years (pack-years)	
	**Values and Rank**									
Austria	28.3 (25.2 - 31.6)	**9**	47.0 (43.6 - 50.4)	**16**	10.6 (9.6 - 11.6)	**11**	10.6 (9.3 - 11.8)	**3**	7.4 (6.3 - 8.4)	**3**
Belgium	19.2 (16.6 - 22.0)	**25**	43.2 (39.8 - 46.7)	**19**	9.9 (9.0 - 10.8)	**16**	7.8 (6.8 - 8.8)	15	4.6 (3.9 - 5.3)	**19**
Bulgaria	36.1 (33.2 - 39.2)	**2**	48.5 (45.4 - 51.7)	**9**	10.9 (10.0 - 11.8)	**8**	8.9 (8.0 - 9.8)	**8**	7.0 (6.2 - 7.8)	**4**
Croatia	35.3 (32.3 - 38.4)	**4**	50.7 (47.5 – 54.0)	**6**	12.0 (10.9 - 13.1)	**4**	11.9 (10.6 - 13.2)	**2**	8.9 (7.8 - 10.1)	**2**
Cyprus	27.5 (23.4 - 32.1)	**12**	44.9 (40.2 - 49.7)	**18**	9.2 (8.1 - 10.4)	**23**	10.2 (8.5 - 11.9)	**5**	6.7 (5.4 - 7.9)	**6**
Czech Republic	28.9 (26.1 - 31.9)	**8**	47.6 (44.4 - 50.7)	**11**	10.7 (9.7 - 11.7)	**9**	8.3 (7.3 - 9.2)	**12**	6.1 (5.3 - 7.0)	**8**
Denmark	18.6 (16.1 - 21.4)	**26**	51.5 (48.0 – 55.0)	**4**	12.6 (11.6 - 13.6)	**3**	8.2 (7.3 - 9.2)	**13**	4.7 (4.0 - 5.4)	**18**
Estonia	23.3 (20.4 - 26.5)	**20**	47.2 (43.8 - 50.7)	**14**	9.5 (8.7 - 10.4)	**20**	5.7 (5.0 - 6.4)	**26**	4.1 (3.5 - 4.8)	**26**
Finland	20.1 (17.5 - 23.0)	**22**	49.2 (45.9 - 52.5)	**8**	10.1 (9.2 - 11.0)	**14**	6.7 (5.9 - 7.4)	**22**	4.1 (3.5 - 4.8)	**26**
France	35.9 (32.6 - 39.4)	**3**	57.5 (54.0 - 60.9)	**1**	13.7 (12.6 - 14.8)	**2**	9.9 (8.8 - 11.0)	**6**	7.0 (6.1 - 7.9)	**4**
Germany	25.8 (23.3 - 28.6)	**16**	46.8 (43.8 - 49.8)	**17**	10.6 (9.8 - 11.5)	**11**	8.8 (7.8 - 9.8)	**9**	5.6 (4.8 - 6.4)	**13**
Greece	36.6 (33.5 - 39.8)	**1**	55.7 (52.4 - 58.9)	**2**	13.9 (12.8 - 15.0)	**1**	13.9 (12.5 - 15.2)	**1**	10.0 (8.9 - 11.2)	**1**
Hungary	26.6 (23.8 - 29.6)	**14**	41.1 (38.0 - 44.4)	**24**	9.5 (8.7 - 10.4)	**20**	8.1 (7.2 - 9.0)	**14**	6.0 (5.2 - 6.7)	**11**
Ireland	19.4 (16.9 - 22.2)	**24**	37.5 (34.4 - 40.7)	**28**	8.3 (7.5 - 9.1)	**28**	6.4 (5.6 - 7.3)	**24**	4.2 (3.5 - 4.8)	**23**
Italy	24.6 (22.0 - 27.5)	**18**	38.3 (35.2 - 41.5)	**27**	10.1 (9.0 - 11.1)	**14**	7.6 (6.7 - 8.5)	**16**	4.9 (4.2 - 5.6)	**16**
Latvia	32.2 (28.4 - 36.3)	**5**	54.9 (50.8 – 59.0)	**3**	11.6 (10.3 - 12.8)	**6**	7.4 (6.2 - 8.6)	**17**	5.7 (4.6 - 6.7)	**12**
Lithuania	29.1 (26.0 - 32.5)	**7**	47.1 (43.7 - 50.6)	**15**	9.6 (8.7 - 10.5)	**19**	5.8 (5.1 - 6.5)	**25**	4.5 (3.9 - 5.2)	**21**
Luxembourg	21.0 (17.3 - 25.3)	**21**	43.2 (38.6 - 47.9)	**19**	8.6 (7.4 - 9.8)	**26**	7.0 (5.7 - 8.4)	**20**	4.6 (3.5 - 5.7)	**19**
Malta	24.0 (19.7 - 28.9)	**19**	43.0 (38.1 - 48.1)	**22**	9.8 (8.4 - 11.1)	**18**	10.3 (8.3 - 12.3)	**4**	6.1 (4.6 - 7.5)	**8**
Poland	29.8 (26.9 - 32.9)	**6**	47.5 (44.2 - 50.8)	**11**	10.7 (9.7 - 11.7)	**9**	8.7 (7.7 - 9.6)	**10**	6.4 (5.5 - 7.2)	**7**
Portugal	25.6 (23.0 - 28.4)	**17**	39.7 (36.7 - 42.7)	**26**	8.8 (7.9 - 9.6)	**25**	5.2 (4.5 - 6.0)	**28**	4.8 (4.1 - 5.5)	**17**
Romania	28.0 (25.2 - 30.9)	**10**	41.9 (38.8 - 45.2)	**23**	9.1 (8.1 - 10.1)	**24**	7.2 (6.2 - 8.3)	**19**	5.5 (4.6 - 6.4)	**14**
Slovakia	26.4 (23.5 - 29.6)	**15**	43.1 (39.9 - 46.5)	**21**	8.5 (7.6 - 9.3)	**27**	5.7 (5.0 - 6.4)	**26**	4.3 (3.7 - 4.9)	**22**
Slovenia	27.9 (25.0 - 31.0)	**11**	47.3 (44.1 - 50.5)	**13**	10.5 (9.6 - 11.4)	**13**	9.4 (8.3 - 10.5)	**7**	6.1 (5.2 - 6.9)	**8**
Spain	27.5 (24.8 - 30.4)	**12**	49.8 (46.6 - 52.9)	**7**	11.6 (10.7 - 12.5)	**6**	8.6 (7.6 - 9.5)	**11**	5.0 (4.3 - 5.7)	**15**
Sweden	7.2 (5.3 - 9.7)	**28**	47.8 (43.5 - 52.2)	**10**	9.9 (8.7 - 11.2)	**16**	6.5 (5.4 - 7.6)	**23**	2.2 (1.6 - 2.8)	**28**
Netherlands	19.5 (17.0 - 22.3)	**23**	51.2 (47.9 - 54.6)	**5**	11.7 (10.7 - 12.7)	**5**	7.3 (6.5 - 8.2)	**18**	4.2 (3.5 - 4.9)	**23**
United Kingdom	17.5 (15.2 - 20.1)	**27**	40.0 (36.9 - 43.2)	**25**	9.3 (8.4 - 10.2)	**22**	6.8 (6.0 - 7.7)	**21**	4.2 (3.6 - 4.8)	**23**

Supplementary Table 8: Five outcomes of smoking burden by socio-demographic characteristics across the 27 EU countries and the UK, Eurobarometer 2017. (N=27,901)

	Prevalence of current smoking	Prevalence of ever smoking	Length of smoking [a]	Pack-years [b]	Discounted pack-years [c]
Unit	(%)	(%)	(years)	(pack-years)	(pack-years)
**Gender**					
Male	30.4	55.2	13.30	11.25	7.00
Female	22.4	38.4	8.55	5.67	4.17
**Age**					
15 - 24 years	29.6	36.0	1.66	0.88	0.87
25 - 34 years	32.9	46.7	5.03	3.20	2.97
35 - 44 years	30.8	49.3	8.97	6.20	5.27
45 - 54 years	31.5	51.9	13.66	10.64	8.42
55 - 64 years	27.1	54.5	17.61	14.95	10.02
65 - 74 years	16.2	45.9	15.79	12.48	6.42
≥75 years	7.9	34.3	12.32	9.68	2.70
**Education (age at completion)**					
≤15 years	22.9	45.5	14.59	11.95	6.76
16–19 years	32.2	52.8	12.69	10.03	7.20
≥20 years	21.8	45.0	9.40	6.60	4.19
Still studying	22.3	28.1	1.44	0.70	0.67
**Difficulty to pay bills**					
Most of the time	42.9	58.8	14.29	12.09	9.66
From time to time	33.4	50.6	11.54	8.91	6.65
Almost never/never	21.7	43.8	10.25	7.72	4.64

Footnote: a-c. Among the entire sample

Supplementary Table 9. Two-level logistic regression and two-level linear regression estimated associations with five outcomes of smoking burden in the 27 EU countries and the UK, Eurobarometer 2017

Socio-demographic characteristics	Outcomes				
	Prevalence of current smoking	Prevalence of ever smoking	Length of smoking	Pack-years	Discounted pack-years
	Odds Ratio (95%CI) [a]		β-Coefficient (95%CI) [b]		
**Gender**					
Male (ref)					
Female	0.57 (0.53 - 0.60) [c]	0.42 (0.40 - 0.44)	-6.4 (-6.8 to -6.1)	-7.5 (-7.9 to -7.2)	-4.1 (-4.4 to -3.8)
**Age**					
15-24 years (ref)					
25-34 years	1.00 (0.86 - 1.16)	1.18 (1.02 - 1.36)	4.0 (3.0 to 5.0)	3.1 (2.1 to 4.2)	3.0 (2.1 to 3.8)
35-44 years	0.87 (0.74 - 1.01)	1.16 (1.00 - 1.34)	7.6 (6.5 to 8.6)	6.1 (5.0 to 7.2)	5.5 (4.6 to 6.4)
45-54 years	0.86 (0.74 - 1.01)	1.22 (1.06 - 1.41)	11.5 (10.4 to 12.5)	9.7 (8.7 to 10.8)	8.1 (7.2 to 9.0)
55-64 years	0.63 (0.54 - 0.74)	1.27 (1.10 - 1.47)	14.2 (13.1 to 15.2)	12.4 (11.3 to 13.5)	8.7 (7.8 to 9.6)
65-74 years	0.31 (0.26 - 0.38)	0.87 (0.74 - 1.03)	11.5 (10.3 to 12.6)	9.2 (8.0 to 10.5)	4.7 (3.7 to 5.7)
≥75 years	0.13 (0.11 - 0.17)	0.56 (0.47 - 0.67)	8.4 (7.1 to 9.6)	6.3 (5.0 to 7.6)	0.9 (-0.1 to 2.0)
**Education (age at completion)**					
≤15 years (ref)					
16-19 years	1.01 (0.92 - 1.12)	1.18 (1.09 - 1.28)	0.1 (-0.5 to - 0.7)	-1.2 (-1.8 to -0.6)	-0.6 (-1.1 to -0.1)
≥20 years	0.61 (0.54 - 0.67)	0.84 (0.77 - 0.91)	-2.4 (-3.1 to -1.8)	-3.7 (-4.4 to -3.1)	-2.8 (-3.4 to -2.3)
Still studying	0.40 (0.32 - 0.49)	0.39 (0.32 - 0.48)	-4.6 (-6.0 to -3.3)	-5.9 (-7.3 to -4.4)	-3.6 (-4.8 to -2.5)
**Difficulty to pay bills**					
Most of the time (ref)					
From time to time	0.76 (0.68 - 0.84)	0.86 (0.78 - 0.95)	-1.6 (-2.3 to -0.9)	-1.9 (-2.7 to -1.2)	-2.1 (-2.7 to -1.5)
Almost never/never	0.52 (0.47 - 0.57)	0.65 (0.59 - 0.72)	-3.4 (-4.0 to -2.7)	-3.8 (-4.5 to -3.0)	-3.9 (-4.5 to -3.4)
**Area of residence**					
Rural (ref)					
Small town	1.06 (0.99 - 1.14)	1.08 (1.02 - 1.15)	0.4 (-0.1 to 0.8)	0.0 (-0.4 to 0.5)	0.2 (-0.2 to 0.6)
Large town	1.11 (1.03 - 1.20)	1.11 (1.04 - 1.19)	0.9 (0.4 to 1.3)	0.5 (0.0 to 1.0)	0.6 (0.2 to 1.0)
**Occupation**					
Employment (ref)					
Unemployment	1.38 (1.24 - 1.54)	1.47 (1.32 - 1.64)	3.1 (2.3 to 3.8)	2.3 (1.5 to 3.1)	2.1 (1.5 to 2.8)
Not working	1.00 (0.90 - 1.11)	1.19 (1.09 - 1.31)	2.5 (1.9 to 3.1)	2.7 (2.0 to 3.3)	1.2 (0.6 to 1.7)
**Marital Status**					
Single households without children (ref)					
Single households with children	1.22 (1.08 - 1.39)	1.36 (1.21 - 1.53)	1.1 (0.3 to 2.0)	0.4 (-0.5 to 1.3)	0.0 (-0.7 to 0.7)
Multiple households without children	0.78 (0.72 - 0.84)	0.99 (0.93 - 1.06)	-0.5 (-1.0 to 0.0)	-0.9 (-1.4 to -0.4)	-1.6 (-2.0 to -1.2)
Multiple households with children	0.67 (0.62 - 0.73)	0.93 (0.87 - 1.01)	-1.2 (-1.8 to -0.7)	-1.3 (-1.9 to -0.8)	-2.0 (-2.5 to -1.6)
**TCS Score [d]**					
Low score (ref)					
Moderate score	0.94 (0.76 - 1.17)	0.95 (0.75 - 1.22)	0.0 (-1.5 to 1.5)	-0.5 (-2.2 to 1.2)	-0.5 (-1.5 to 0.6)
High score	0.69 (0.54 - 0.87)	0.94 (0.72 - 1.23)	0.0 (-1.7 to 1.7)	-0.5 (-2.4 to 1.5)	-1.2 (-2.3 to 0.0)

Footnote:
a. Two-level logistic regression adjusted for all variables included in the table
b. Two-level linear regression adjusted for all variables included in the table
c. 95% confidence interval
d. TCS=Tobacco Control Scale

Supplementary Figure 1: Map of the prevalence of ever smoking and length of smoking in the entire population across the EU countries, Eurobarometer 2017

D. Prevalence of Ever smoking **E. Length of smoking**

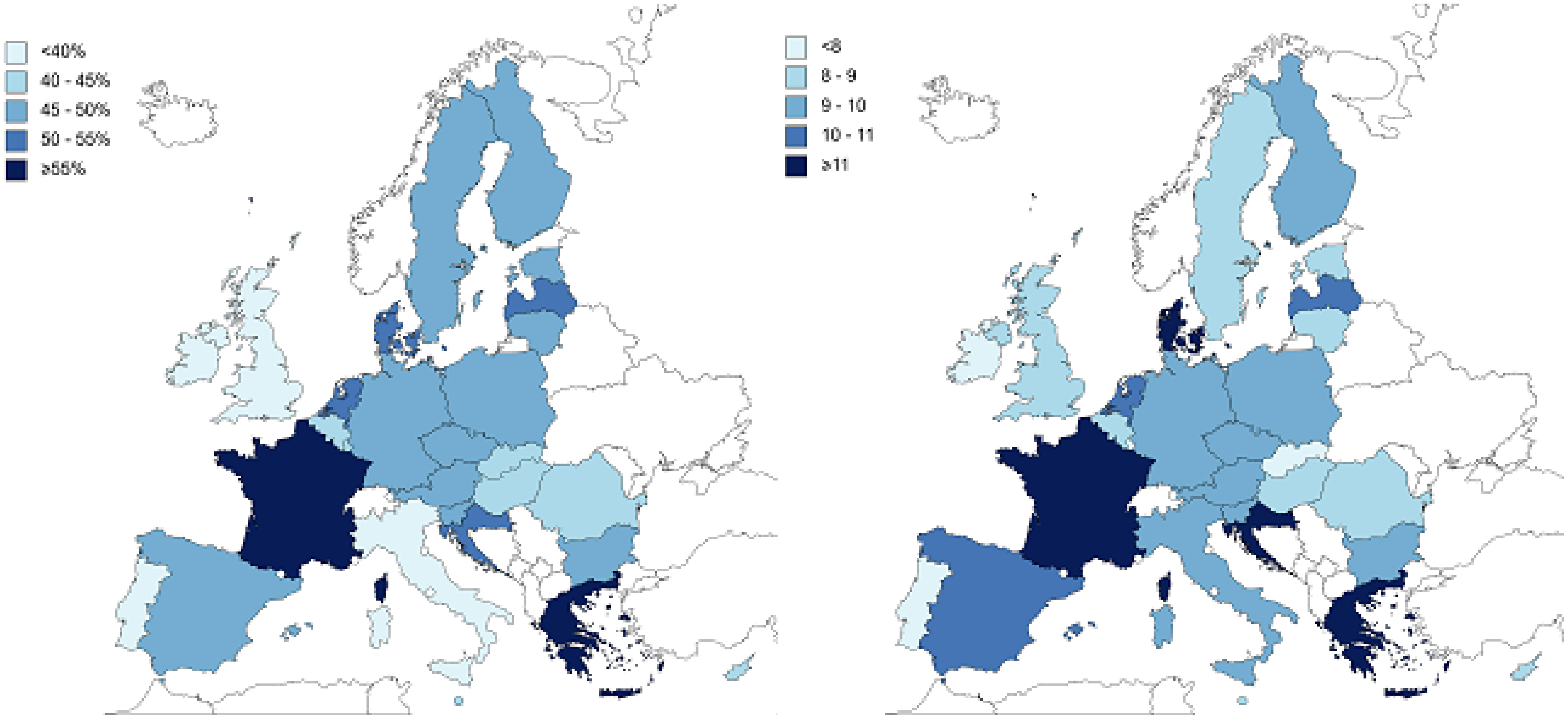

Supplementary Figure 2: Scatterplots of each indicator across the EU countries, Eurobarometer 2017

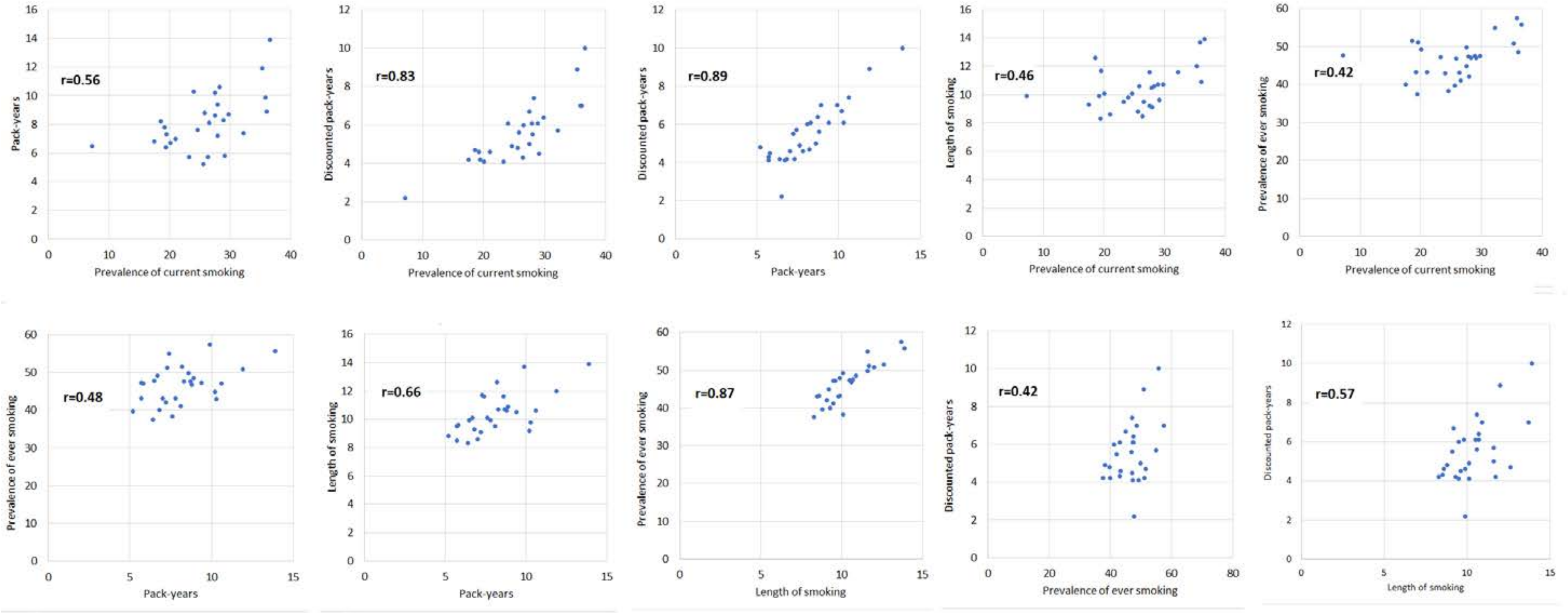

Supplementary Figure 3: Five indicators of smoking burden by socio-demographic characteristics across the 27 EU countries and the UK

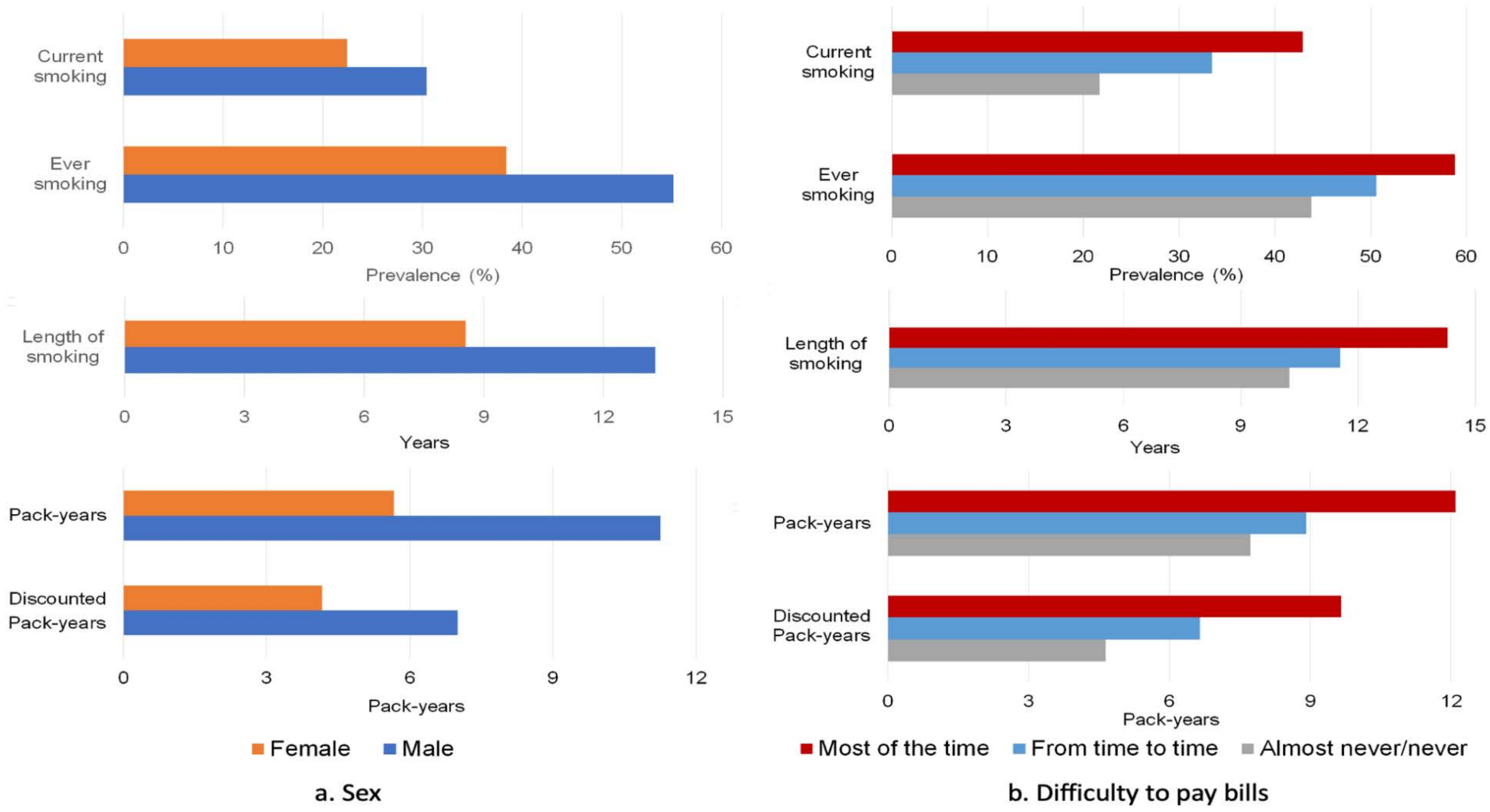

© 2022 Teshima A. et al.
